# How Spatial Epidemiology Helps Understand Infectious Human Disease Transmission

**DOI:** 10.3390/tropicalmed7080164

**Published:** 2022-08-02

**Authors:** Chia-Hsien Lin, Tzai-Hung Wen

**Affiliations:** 1 Department of Health Promotion and Health Education, National Taiwan Normal University, Taipei City 10610, Taiwan; 2 Department of Geography, National Taiwan University, Taipei City 10617, Taiwan; wenthung@ntu.edu.tw

**Keywords:** hot spot, neighbourhood effect, spatial-temporal analysis, spatial epidemiology, spatial heterogeneity, visualization, spatial pattern, spatial regression

## Abstract

Both directly and indirectly transmitted infectious diseases in humans are spatial-related. Spatial dimensions include: distances between susceptible humans and the environments shared by people, contaminated materials, and infectious animal species. Therefore, spatial concepts in managing and understanding emerging infectious diseases are crucial. Recently, due to the improvements in computing performance and statistical approaches, there are new possibilities regarding the visualization and analysis of disease spatial data. This review provides commonly used spatial or spatial-temporal approaches in managing infectious diseases. It covers four sections, namely: visualization, overall clustering, hot spot detection, and risk factor identification. The first three sections provide methods and epidemiological applications for both point data (i.e., individual data) and aggregate data (i.e., summaries of individual points). The last section focuses on the spatial regression methods adjusted for neighbour effects or spatial heterogeneity and their implementation. Understanding spatial-temporal variations in the spread of infectious diseases have three positive impacts on the management of diseases. These are: surveillance system improvements, the generation of hypotheses and approvals, and the establishment of prevention and control strategies. Notably, ethics and data quality have to be considered before applying spatial-temporal methods. Developing differential global positioning system methods and optimizing Bayesian estimations are future directions.

## 1. Introduction

Infectious diseases in humans can be directly or indirectly transmitted in time and space. For example, influenza and pertussis are considered as diseases that can be directly transmitted when aerial droplets produced through sneezing or coughing from an infectious person spread over a short distance to a susceptible person [1]. Therefore, the physical distance between humans must be short enough for successful transmissions. On the other hand, indirect transmissions refer to diseases that are spread from person-to-person via biotic animals or abiotic components (e.g., water and soil) [1]. This mode of transmission emphasizes the environments shared by susceptible humans, infectious animals, or contaminated objectives.

Spatial dimensions are crucial when considering the management of infectious diseases. From an epidemiological point of view, an outbreak of an epidemic is defined by any temporally unusual increase in numbers of case-patients in a localized area [2,3]. Recent development of sophisticated statistics and advanced computerized software, such as geographical information systems, can provide health authorities more spatially-relevant information, such as the range and direction of diseases spreading or the hot spot locations of diseases, and hence, control measures could be more effective. For a better understanding and management of infectious diseases, spatial approaches are necessary to be further integrated into the implementation of prevention and control measures against epidemics.

There are two types of spatial data commonly used in health research. Point data are raw data which include health events such as incidences and deaths, health facilities such as hospitals and clinics, and physical objectives such as dump sites and mosquito breeding sites. Contrary to point data (i.e., individual data), aggregate data are summaries of individual points by time or by space. Aggregate data, such as incidence rates and fatality rates in countries or population densities in administrative units, were commonly used to explore the relationships between disease outcomes and potential risk factors, geographically.

**Figure 1:**
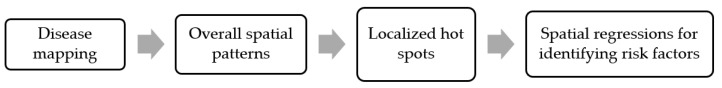
Four main sections in order in this review.

The objective of this review article is to provide a brief overview of the common spatial or spatial-temporal statistical methodologies and their applications for better understanding of disease transmission. This article is classified into four main sections (as seen in Figure 1 below). The first part covers spatial visualization of infectious diseases which illustrates the distribution of health outcomes data and relevant information on maps. The second section lists spatial statistical methods for the identification of overall spatial clustering patterns of diseases, and the third section provides the methods of localized hot spots. The last section outlines two geographical properties of disease data, namely the neighbourhood effect and spatial heterogeneity. Moreover, this section reviews common spatial regression techniques available for dealing with the two geospatial properties. The summary of four main sections and their characteristics and examples is shown in Table 1.

## 2. Disease Mapping

### 2.1. Location Mapping

Mapping disease locations is the most straightforward and earliest spatial approach in management of infectious diseases. This type of map used point data. Seaman drew the location of yellow fever deaths and the waste sites on the map of New York in 1798 [4,5]. Seaman further described the observed geographical relationships between case deaths and the dump sites [4,5]. Another well-known mapping study was conducted by John Snow. In 1854, John Snow used dots to represent the location of cholera cases on the London road network map. The map revealed that cholera death cases were located around the water pump [6]. In both cases, dot distribution maps were presented.

Mapping disease locations have several advantages and limitations. One of its advantages is that it enables researchers to quickly observe and describe spatial distributions or spatial densities of diseases [43]. In addition, visualizing disease distributions could provide hypotheses to conduct epidemiological investigations [4,6,7]. With regard to the limitations, patients’ addresses may be difficult to obtain due to privacy reasons, especially for infectious diseases. It is also difficult to know whether the patient distribution is random or not. Finally, a disease location map is difficult to reflect the spatial distributions of underlying overall populations or susceptible populations [8].

### 2.2. Surface Mapping

In addition to location mapping, surface maps, using aggregate data, have often been employed in various epidemiological disease studies. Commonly applied surface maps include choropleth maps, which present summary statistics in area units with colours. For example, in the filariasis study in India from 2004 to 2007, Upadhyayula et al. produced the prevalence of infection rate, mosquito per man hour, infectivity rate, and microfilaria rate in each surveyed village in four choropleth maps, respectively [9]. Based on the maps, the authors concluded that although intervention programmes had been implemented, the microfilariaemia rate was still at a concerning level in India. Another example was the 2009 Q fever outbreak investigated by Soetens et al. in the Netherlands. They drew choropleth incidence maps with dissimilar spatial scales and classification methods (i.e., Jenks’ natural breaks and the quantile) and found that the choropleth Q fever incidence map was sensitive to dissimilar spatial scales and classification methods [8].

Choropleth maps include certain advantages and disadvantages. One of the advantages of choropleth maps is that disease statistics, such as incidence rates, can be easily and comprehensibly visualized on one map. It also helps people to understand the situations in their living areas compared to other areas. Finally, the maps can be applied for all administrative scales. However, as choropleth maps use a generalized summary, it may hide important factors associated with diseases, such as demographic characteristics (e.g., sex and age) and socioeconomic statuses (e.g., income and education) among individual patients. Moreover, choropleth maps are prone to different spatial scales and classification methods [8]. This characteristic may provide the map readers with misleading interpretations when changes in scales or classifications are made.

The misleading interpretations can also happen when small numbers of cases or deaths in sparsely populated areas [10]. The disease rates are, therefore, extremely high. To reduce this type of bias, Bayesian smoothing methods provide solutions.

Bayesian mapping approach is to apply Bayesian probability models to quantity and smooth estimations [10,11]. Applying Bayesian approaches show less biased than choropleth maps in local risk estimations because the estimations are employed by local neighbourhoods [10]. Bayesian risk mapping was widely applied in infectious diseases, including dengue [12,13], influenza [14,18], and tuberculosis [15,17]. However, one of the problems of Bayesian estimations is computational difficulties [16].

Another smoothing method is kernel density estimation (KDE) which works by identifying “dense” points and allowing those dense points to be visualized as a smoothly surface on the map [19]. Telle et al. used KDE to detect the local intensity of dengue cases in the endemic urban area, Delhi, India between 2008 and 2010 [20]. As a result, the locations of the high concentration of cases were presented differently between 2008 (west, central, and east Delhi) and 2009 (central, east, and south Delhi).

Disease mapping techniques provide an effective approach to describe and observe disease patterns spatially. Nevertheless, mapping diseases cannot objectively quantify spatial or spatial-temporal patterns in health outcomes. Therefore, spatial statistics are used for providing an in-depth understanding of disease spatial phenomena. Nowadays, thanks to the improvements of statistical methods and advanced technologies in computing performance, there are new possibilities regarding the convey and analysis of disease spatial data [33,44,45].

## 3. Overall Spatial Patterns

Disease overall clustering is the observed disease distribution having a significant aggregated pattern compared to a hypothetical random distribution over an area. In order words, overall clustering refers to the observed pattern over an area that is not due to chance [43,46]. Several methods have been developed for testing overall clustering. Those approaches can apply to either point data or aggregate data.

### 3.1. Statistical Tests of Overall Clustering for Point Data

In general, statistical tests of overall clustering patterns for spatial point events are based on the distances between pair-points. Several statistical methods have been developed, such as the nearest neighbour ratio [47], Cuzick and Edwards’ test [48], and Ripley’s K function [49], and there were many examples of these methods in communicable disease studies. Guo et al., used the nearest neighbour ratio method (i.e., the ratio of the observed average distance among cases to the expected average distance among the same number of cases) to assess the clustering degree of human rabies infection in China [21]. It was observed that an annual increase in the nearest neighbour ratio from 2005 to 2009, which indicated that there was an increase in clustering degree of rabies infection in China. In Brazil, human visceral leishmaniasis (HVL) is an emerging anthropozoonosis and Bermudi et al. applied Ripley’s K function to investigate the clustering of autochthonous HVL cases in São Paulo during three study periods: 1999–2004, 2005–2009, and 2010–2015 [22]. K function showed that during the period of 1999–2004 and 2005–2009, HVL presented a clustering pattern within 2000 m and 3000 m, respectively. This finding implied that in addition to vector fly distance, other factors, including reservoirs and human movements, could be involved in HVL cases spatial distribution. Later, instead of investigating HVL human, Silva et al. examined domestic dogs, the main reservoir of leishmania parasites, in another state of Brazil [23]. They defined case dogs with a higher immunofluorescence antibody test reaction (≥1:40 titer), and those with lower titers as controls. With the Cuzick and Edwards’ test, the finding showed that only the first and fourth nearest neighbors demonstrated significant clustering patterns in Piauı, Brazil. Lai et al. applied the nearest neighbour analysis based on the R scale to identify the clustering of severe acute respiratory syndrome (SARS) in Hong Kong from 15 February to 22 June 2003 [24]. Except for weeks with too few cases (*n* < 25), SARS cases showed significant clustering patterns over 16 weeks.

### 3.2. Statistical Tests of Overall Clustering for Aggregate Data

Spatial autocorrelation statistics are the degree of similarity among the observation values at spatial locations. Positive spatial autocorrelation indicates that neighboring values are geographically similar. In other words, high value areas tend to be close to high value areas, and low value areas tend to be near low value areas on the maps. One of the key determinants for measuring spatial autocorrelation is the spatial neighbourhood. Neighbourhoods can be defined by distance, contiguity, or other characteristics [26,43], and spatial relationships among neighbourhoods will be formed as the weight matrix [50]. Consequently, if one uses distance to define neighbours, then, the closer the areas, the larger the weight matrix [51].

Statistics of quantifying spatial autocorrelations, such as Moran’s I and Geary’s C have been widely used [50,51,52,53,54]. Other graphics and diagrams, such as Moran’s scatterplot, spatial correlograms, and semivariogram, were also commonly employed in spatial epidemiological studies [27,28,43].

Spatial autocorrelation in infectious diseases has been frequently recognized [24,26,29,30,31]. For example, the dengue study conducted by Lin et al. showed that by applying Moran’s I, dengue incidence rate at village-level had a significant clustering pattern annually in Kaohsiung, Taiwan from 2003 to 2009 [29]. Hamric et al. investigated yellow fever in the Americas (WHO region) from 2000 to 2014 and found that case-positive countries had significant (*p* < 0.001) positive, spatial autocorrelation (I = 0.02) [30]. Lai et al. investigated SARS outbreak in Hong Kong from February to June 2003 [24]. They employed Moran’s I with the polygons having a common border or corner as neighbours, and the results showed that infection rates were significant clustering on 12 prototypical days over 16 weeks. The COVID-19 study conducted by Kang et al. applied Moran’s I with different definitions of neighbourhoods (i.e., adjacency, distance, population, population density, number of doctors and hospitals, and number of medical beds) for daily new confirmed cases in China in 2020 [26]. Among six definitions, five showed the existence of positive, significant, spatial autocorrelation from 22 January 2020. This study implied that the neighbourhood types could vary the study conclusions. In South Korea, another COVID-19 study conducted by Kim and Castro investigated the spatial distribution of the incidence rate in each district from 20 January to 31 May 2020 [31]. The Moran’s I coefficient showed that incidence rate of COVID-19 had a significant strong spatial autocorrelation (I = 0.78, *p* < 0.001) in South Korea.

## 4. Localized Hot Spots

Local spatial estimations enable us to identify locations of disease clusters (i.e., hot spots). By being aware of the hot spots’ locations, health authorities may have better ideas for identifying resources efficiently. Furthermore, people could imply that environment factors could have an impact on disease hot spots based on the identified clusters and physical features such as markets and rivers on the maps. Similar to the overall pattern, different approaches for point data and aggregate data were developed.

### 4.1. Detections of Localized Clusters for Point Data

The methods used to detect localized point clusters are commonly density-based such as KDE. Other methods based on machine learning are used for cluster identifications, such as density-based spatial clustering of applications with noise (DBSCAN), hierarchical density-based spatial clustering of applications with noise, and ordering points to identify the clustering structure; these methods were developed based on different algorithms.

In Hong Kong, Lai et al. applied a modified kernel approach which was adjusted for population density for 2003 SARS [24]. As the kernel approach was adjusted for population, the identified SARS hot spots represented the populations at risk. From 2005 to 2011, Guo et al. investigated the distribution of human rabies in space and time in China [21]. They identified 480 hot spots from 17,760 human rabies cases by spatiotemporal DBSCAN method. Among all hot spots, over 80% were detected in the first three years (i.e., 2005–2007). A modified spatiotemporal DBSCAN (MST- DBSCAN) approach proposed by Kao et al. was used to detect the 2014 dengue hot spots in Kaohsiung, Taiwan [32]. By using MST-DBSCAN, Kao et al. classified dengue cases into 10 cluster evolution types based on the sources of infection and transmission clusters and successfully visualized different evolution type clusters on the map. De Ridder et al., also applied a MST- DBSCAN approah to investiagte laboratory confirmed COVID-19 patients, Geneva, Switzerland from 26 February 16 April 2020 [33]. The findings of space-time clusters revealed that the first local hot spots started in the areas with the highest numbers of people per room.

### 4.2. Detections of Localized Clusters for Aggregate Data

To detect localized clusters, many methods based on different spatial characteristics, such as spatial dependency [55], spatial intensity [34,35,56,57], or the likelihood ratios [22,31,36,37,58] have been developed.

The local version of spatial autocorrelation, which is also called LISA, local Moran’s I, or Moran’s Ii, is the most commonly used [55]. For example, Lin et al. conducted a dengue incidence rates study in urban Kaohsiung, Taiwan from 2003 to 2009 and found unpredictable locations of hot spots detected by LISA from one year to another [29]. A study of yellow fever cases in the Americas (2000–2014) conducted by Hamric et al. at county-level showed that the LISA statistic identified locations of hot spots mainly in Peru and Colombia [30]. Alene et al. analysed the prevalence of poor tuberculosis treatment outcomes (i.e., lost to follow up, treatment failure, and death) at district-level in Ethiopia from 2015 to 2017 [35]. The LISA identifying the hot spot areas were mainly in northeast and west of Ethiopia.

Hot spots can also be identified by Getis-Ord Gi* statistic which measures the *intensity* of high values [34,35,56,57]. In the study conducted by Alene et al., Gi* statistic was applied to identify locations of hot spots for the prevalence of poor tuberculosis treatment outcomes areas [35]. According to the findings, the locations of hot spot regions identified by Gi* statistic were similar to those identified using the local Moran’s I. Hinman et al. used the local Getis-Ord Gi statistic in a way to understand the typhoid fever in Washington, D.C. from 1906 to 1909 [34]. They found that the locations of hot spots differed from one year to another.

Scan statistics, a likelihood-based approach, is another widely used model-based statistics to identify the locations of clusters for aggregate data [22,31,36,37,58]. Scan statistics include pure spatial/space-time Bernoulli and Poisson models, and space-time permutation for the early detection of disease outbreaks. One of the examples with Poisson models was that Bermudi et al. applied space-time scan statistics to identify clusters of the high risk areas for HVL in respect of spatial and temporal dimensions in Brazil [22]. As a result, two spatial clusters of high-risk area were identified from 1999 to 2015, and three spatiotemporal clusters (relative risk: 10.3, 5.4, and 3.3 in 2001–2003, 2003–2004, and 2002–2008, respectively) were found. Another example was in South Korea, Kim and Castro applied scan statistic with a Poisson model to explore spatiotemporal clusters of COVID-19 cases by district in 2020 [31]. Using the scan statistic, they identified 12 significant COVID-19 clusters without spatial overlaps. Regarding space-time permutation models, Coleman et al. used space-time permutation and the Bernoulli purely spatial models to understand malaria spatial and temporal clusters in seven towns of Mpumalanga, South Africa from 2002 to 2005 [36]. Space-time malaria clusters were detected between 2004 and 2005 in two out of the seven towns by the circular scan statistic. As for Bernoulli models, in Western Kenya, Brooker et al. applied the spatial scan statistics to identify malaria case clusters during a 10-week malaria outbreak in 2002 [37]. They found malaria case households in the detected clusters located at lower altitudes than those outside the identified spatial clusters.

Notably, in the study area, significant localized hot spots can be detected but not the significant overall clustering. This can be demonstrated by Wheeler’s childhood (age 0–14) leukemia study in Ohio, USA from 1996 to 2003 [25]. In Wheeler’s study, spatial overall clustering was tested by K function, Cuzick and Edwards’ method, and the kernel intensity function ratio summary, and all three methods showed no statistically significant overall clustering. However, localized hot spots were detected by kernel intensity function.

Clustering analyses can provide answers to what overall disease spatial patterns are present in the area. Cluster detection approaches, on the other hand, can be used to identify localized disease hot spots. However, these analyses cannot identify risk factors that are geographically associated with health outcomes. Regression analysis is, therefore, a feasible method that can be employed to identify such risk factors.

## 5. Spatial Regressions for Identifying Risk Factors

Conventional non-spatial regression methods are not suitable for spatial disease data for two reasons: spatial dependency and spatial heterogeneity. Spatial data are often dependent on each other due to nested structures in data [43]. Nested structures mean, for example, that patients are nested in households, households are nested in communities, communities are nested in districts, and districts are nested in cities. Depending on the disease type, patients could be similar in certain nested levels due to their shared environments [43]. This similarity causes data dependency. The spatial data dependency is against one of the main requirements for applying non-spatial regression models, such as ordinary least squares (OLS) and Poisson models. In addition to spatial dependency, disease data are often heterogeneous geographically. Spatial heterogeneity refers to data that have dissimilar distributions of events, concentrations of events, or relationships over space [59]. For example, according to Lin and Wen’s study, the dengue incidence rate was associated with population density in one location but not in another location [41]. This property also does not fit the requirement of traditional regression models. Therefore, due to the above reasons, spatial data analysed by non-spatial regression methods are not appropriate [60].

### 5.1. Spatial Neighbourhood Effect

The spatial neighbourhood effects in health sciences are the phenomena that the health outcome in one location is affected by the health outcomes in its spatial neighbourhoods. That is, the health outcome is spatial dependent.

Different from non-spatial regression models, spatial regressions account for spatial dependency. In other words, spatial regression approaches can handle situations such as the incidence rate in one district being influenced by the incidence rates in its surrounding districts. Spatial regression methods, such as spatial lag model (SLM) and spatial error model (SEM), by using maximum likelihood estimations were widely applied [38,39]. Moreover, Bayesian spatial models, such as conditional autoregressive (CAR) model, by Markov chain Monte Carlo methods were in accounting for spatial dependency in infectious diseases [40].

Some examples of the use of SLM, SEM, and CAR were showed in the following epidemiological literatures. In Chaurasia’s study, they investigated diarrhea prevalence rate associated with socio-demographic, socio-economic, and environmental factors at district-level among children aged from five to 10 in India from 2015 to 2016. Chaurasia et al. found that four out of 10 factors, including spring season and open defecation, were identified as significant risk factors by applying OLS, SLM, and SEM [38]. They further concluded that due to the smaller Akaike information criterion (AIC) values, both spatial SEM and SLM performed better than non-spatial OLS (AIC 3712, 3720, and 3818 for SEM, SLM, and OLS, respectively). In Mollalo’s study, OLS, SLM, and SEM approaches were used to analyse the COVID-19 incidence rates at county-level across the United States from 22 January to 9 April 2020 [39]. Based on adjusted R^2^, Mollalo et al. concluded that SLM and SEM still performed better than OLS. Almeida et al. examined the 2001–2002 dengue epidemic in 154 neighbourhoods of the municipality in south-eastern Brazil [40]. To assess the association between the socioeconomic factors and dengue incidence rates, Almeida et al. applied a CAR model that allows spatial dependency of incidence rates in each nearby neighbourhood. Through this approach, they identified that the percentage of households connected to the general sanitary network was the significant risk factor of the average incidence rate of dengue. This study highlighted the importance of improvements in environmental sanitation.

### 5.2. Spatial Heterogeneity

Spatial regression models, such as geographically weighted regression (GWR) and multiscale GWR (MGWR) models, can deal with heterogeneity in spatial data [61,62,63,64]. Both methods handle non-stationary spatial relationships between dependent and independent variables by estimating coefficients in each data location.

Tsai and Teng applied GWRs to a study of risk factors for dengue incidence rates at township-level in Taiwan from 2009 to 2011 [28]. The findings showed that Breteau indices of *Ae. albopictus* had no significant impacts on indigenous dengue incidence rates in overall Taiwan except the southwest regions. Mollalo et al. applied GWR and MGWR to model COVID-19 incidence rates in continental United States in 2020. Both methods showed that by including the percentage of black female population, median household income, percentage of nurse practitioners, and income inequality in the models, over 67% of the variances in the COVID-19 incidence rates could be explained [39]. Urban and Nakada investigated the number of COVID-19 deaths (both confirmed and suspected) in 96 districts in the city of São Paulo, Brazil from March to June 2020 [42]. By applying GWR, they found geographically heterogeneous relationships between the number of deaths and four demographic and socioeconomic variables (i.e., persons aged 60 or above, population density, average people per household, and Municipal Human Development Index score). For instance, in the south-western districts of the city, the number of deaths was strongly associated with people aged 60 or above but less associated with other three variables.

## 6. Discussion

Understanding spatial-temporal variations in the spread of infectious diseases in humans have three positive impacts on future control strategies, as discussed below:

First, understanding of spatial epidemiology improves sensitivity and representativeness in the communicable disease surveillance systems. One of the purposes of infectious surveillance systems is to detect epidemics in the early stage for timely interventions [65]. If we know the identified risk objectives in space (e.g., rivers), the characteristics of risk objectives (e.g., water quality) could be integrated into disease surveillance systems in order for the systems to exhibit better capacities to detect occurrences of communicable diseases. Another important attribute of a surveillance system is that the system needs to be representative.

Representativeness of surveillance systems means data in systems can correctly reflect disease distributions in space and time [66]. If the spatial risk factors, such as communities with low socioeconomic status or densely populated areas, have been identified, the health authorities can perform additional surveys in these risk areas in order to identify potentially-infectious patients who were not detected in the original system. Therefore, patients’ data in surveillance systems are more representative by including spatial information.

Second, investigators can generate and prove hypotheses by applying spatial-temporal approaches in outbreak investigations. For example, by disease mapping, investigators can observe patients with diarrhoea who live along a river, and hence, investigators can hypothesize that the closer people live around the river, the easier it is to experience diarrhoea. To prove this hypothesis, investigators may collect spatial data, such as the distances between households and the investigated river, as well as temporal data, such as onset date. Then, they can examine the spatial associations between the characteristic features of the patients and the river, or conduct spatial regressions to identify if the distance is a risk factor of diarrhoea occurrences.

Last, understanding spatial-temporal transmission phenomena helps to make prevention and control strategies. Taking COVID-19 infection as an example, studies showed that transmission rates of SARS-CoV-2 were the combinations of space-time factors, including exposure periods, airflow patterns and physical distances [67,68,69,70,71]. To reduce the transmission rates, policymakers can manipulate either spatial or temporal factors in the communities.

Before applying space-time methods in infectious disease studies, researchers have to consider some issues. One of the main issues is the ethical concern. A study conducted by de Jong et al. discussed two ethical considerations in mapping infectious diseases: patient privacy, and balance between patient and community benefits [72]. Authors emphasized that ethical standards should especially ensure that the same standards for vulnerable groups, such as low-income populations, were met. In addition, the health authorities should think about how to communicate information, such as patient movements and hotspot locations, to the populations.

Another issue is the representativeness and accuracy of spatio-temporal data. Smartphones are often used as tracking devices in measuring people movements [73,74]. However, not everyone has a smartphone. Populations, such as children under five years of age or people over certain ages may not have smartphones. Therefore, smartphone-tracking data do not include those populations. Obtaining spatial-temporal data from non-smartphone users needs other approaches, such as interviews or the use of questionnaires. In terms of smartphone users, studies showed that global positioning system (GPS) positioning accuracies were different by phone brands and with deviations from one to ten m [73,75,76,77]. Moreover, GPS positioning abilities would be reduced by the environments, such as buildings and weather. Therefore, using space-time data from smartphones via GPS also has its limitations.

Future developments in analysing and predicting the spread of infectious diseases have two directions that can be improved: statistical methods and capacity of geopositioning technologies. Bayesian mapping and analyses are known by their intuitive approaches of combining prior data. However, the computation difficulties are the challenges. Therefore, strategies of optimizing Bayesian estimating processes are needed. Regarding geopositioning technologies, currently distances between people cannot be correctly measured by phones via GPS positioning. Consequently, the development of differential GPS methods is, therefore, needed in order to better track diseases.

## 7. Conclusions

The studies of infectious human diseases distributions in the spatial or spatial-temporal perspectives will provide additional information compared to information obtained only from the standpoints of temporal perspectives. Descriptive graphical representations provide audiences with straightforward and intuitive impressions on disease patterns. Various spatial statistical methods have been developed to examine disease patterns or hot spots. Spatial regressions account for neighbourhood effects or for spatial heterogeneities can be applied to further determine the possible factors associated with identified hot spots. The applications of spatial approaches to infectious diseases allow policymakers to better allocate intervention resources against disease outbreaks. Additionally, it helps in increasing authorities’ awareness of the environmental or demographic risk factors of infectious diseases. Therefore, developing advanced methodological framework, such as simulation, for incorporating spatial-temporal epidemiological data to examine cause-effect relationships between exposure and infectious diseases warrants further investigation.

## Figures and Tables

**Table 1:** Summary of themes, categories, data types, and examples in this article.

Spatial Theme	Category	Data Type	Examples
Disease Mapping	Location mapping	Point	[4,5,6,7,8]
	Surface mapping	Aggregate	[8,9,10,11,12,13,14,15,16,17,18,19,20]
Overall patterns	Clustering	Point	[21,22,23,24,25]
		Aggregate	[24,26,27,28,29,30,31]
Localized hot spot	Cluster	Point	[21,24,25,32,33]
		Aggregate	[22,25,29,30,31,34,35,36,37]
Identifying risk factors	Neighbourhood effect	Aggregate	[38,39,40]
	Heterogeneity	Aggregate	[28,39,41,42]

## Data Availability

All data are included in this manuscript. There are no other data involved in this study.
